# Therapeutic Benefit of Intravenous Administration of Human Umbilical Cord Blood- Mononuclear Cells Following Intracerebral Hemorrhage in Rat

**Published:** 2012

**Authors:** Masoumeh Seghatoleslam, Mehdi Jalali, Mohammad Reza Nikravesh, Mahmoud Hosseini, Daryoush Hamidi Alamdari, Alireza Fazel

**Affiliations:** 1 *Department of Anatomy and Cell Biology, Faculty of Medicine, Mashhad University of Medical Sciences, Mashhad, Iran*; 2 *Department of Physiology, Faculty of Medicine, Mashhad University of Medical Sciences, Mashhad, Iran*; 3 *Department of Biochemistry, Faculty of Medicine, Mashhad University of Medical Sciences, Mashhad, Iran*

**Keywords:** Functional recovery, Human umbilical cord blood- mononuclear cells (HUC-MCs), Inracerebral hemorrhage (ICH), Striatum

## Abstract

**Objective(s):**

Human umbilical cord blood (HUCB) is now considered as a valuable source for stem cell–based therapies. Previous studies showed that intravascular injection of the HUCB significantly improves neurological functional recovery in a rat model of intracerebral hemorrhage (ICH). In the present study, we hypothesize transplanted HUCB derived mononuclear cells (UC-MCs) can decrease injured volume and also ameliorate neurological function in ICH rats.

**Materials and Methods:**

Experimental ICH was induced by intrastriatal administration of collagenase in rats. One day after surgery, the rats were divided into 3 groups to receive intravenously either BrdU positive human UC-MCs [(4×10^6^ and 8×10^6 ^cells in 1 ml saline, n=10 respectively) as treated groups] or the same amount of saline [as lesion group (n=10)]. There was also one group (control) that received only vehicle solution of collagenase. The animals were evaluated for 14 days with behavioral tests. Transplanted UC-MCs were detected by immunohistochemistry. Histological data and scores of functional tests were analyzed using ANOVA. Cellular co-localization of BrdU+ cells in the histological slides was determined by software Image J.

**Results:**

Intravenously transplanted UC-MCs migrated selectively to the hematomal area and reduce injured volume. The UC-MCs transplanted groups showed better performance on functional tests after 2 weeks compared with the lesion and control groups (*P*< 0.05). There was no difference in the functional recovery and injured volume improvement between the 2 treated groups.

**Conclusion:**

Intravenously transplanted UC-MCs accelerate neurological function recovery of ICH rat and diminish the striatum lesion size. Thus these cells may provide a potential cell candidate for cell-based therapy in ICH.

## Introduction

Intracerebral hemorrhage (ICH), which results from the spontaneous rupture of an intracranial vessel, is a subtype of stroke with high morbidity and mortality rate of about 15% of all deaths from stroke and it happens frequently as a major complication of thrombolytic therapy for acute ischemic stroke (1) and it leaves many survivors disabled (2). The striatum component of the basal ganglia is the most common site for intracerebral hemorrhage and accounts for about one-third to one-half of all ICHs (3).

Despite several advantages in stroke care and therapy, only a small number of acute stroke patients (3%) receive specific therapy (4) and thus, the prognosis of patients after ICH is poor often much worse than that of patients with ischemic strokes of similar size. Currently, there is no available medical therapy for patients with ICH, and supportive care or invasive neurosurgical evacuation of hematoma in selective patients is all that can be done (5).

Over the past years, cell transplantation has been proposed as a potential approach to the treatment of neurological disorders (6, 7). A number of different cell sources have been used successfully in various experimental models of stroke (8). However, ethical considerations and limited availabilities restrict the application of several cell populations. One cell population of interest consists of human umbilical cord blood (HUCB) cells that are a source of hematopoietic stem/progenitor cells (HSC) (9), mesenchymal stem/progenitors (MSC) (10) and endothelial progenitors (11). Since 1989, when the first successful umbilical cord transplantation was reported in a child with Fanconi anemia, thousands of transplants have been carried out, as a therapy for certain malignant and nonmalignant hematological disorders (12). Moreover, in the last few years, preclinical studies have shown that HUCB injected systemically in the acute phase of animal models of stroke have a therapeutic effect. These cells can reduce the area of brain infarction (13) and the inflammation (14), thus, increase the regenerative capacity of the brain (15), and consequently improves the behavioral recovery (16). Additionally, HUCB cells can secrete numerous neurotrophic factors (17). Presently, it is not known whether any of these stem/progenitor cells contribute to get over neurologic deficits seen in animals treated with UCB. In addition, despite the fact that several groups have reported beneficial effects of HUCBC administration after ischemic stroke, there has been a shortage of study of the therapeutic benefit of UCBCs on hemorrhagic stroke.

However, in other cell therapy studies related to brain disorder, researchers prescribed the stem cells to animals' intracerebraly and only a few of them preferred to deliver the cells intravenously. Whereas, systematic delivery of these kinds of cells in experimental models can help to find better clinical approaches in the ICH patients, in this study stem cells were administrated intravenously. Also since mononuclear cells were obtained from human umbilical cord blood (HUCBC) as a subtype of HSC, they are easy to obtain, ethically unproblematic, available for allogeneic approaches and suitable candidates for cell therapies (18).

Hence, the aim of the present study was to answer the question whether intravenously delivered HUCB-derived mononuclear cells could decrease injured volume of ICH in rats and lead to improve neurological function or not?

## Materials and Methods

***Chemicals***

Collagenase type IV, bromodeoxyuridine (BrdU) and antibodies were purchased from Sigma Chemical Co and other materials were provided from Merck Company.

***Animal model of intracerebral hemorrhage (ICH)***

Forty male Wistar rats, weighing 250–300 g each, were used throughout the study. All of them were housed in the same room under a constant temperature (22±2 °C) and illuminated 7:00 a.m. to 7:00 p.m., with food pellets and water available *ad libitum*. Animal handling and all related procedures were carried out in accordance with Mashhad University of Medical Sciences, Ethical Committee Acts.

The animals were anesthetized by 100 mg/kg/bw ketamine hydrochloride and 4mg/kg/bw xylazine intraperitoneally (i.p.) and placed in a stereotaxic apparatus. A midline incision was made through the scalp to expose the skull. A 28-gauge needle was implanted into the striatum at coordinate’s 0.2 mm posterior and 3.0 mm lateral to bregma, depth 6.0 mm ventral to the surface of the skull (19, 20) and 1.0 µl of sterile saline containing 0.24 U of bacterial collagenase IV was infused over a period of 5 min by a Hamilton syringe connected to needle into the striatum. However, for animals in control group 1.0 µl of sterile saline only was infused. At the end of infusion, the needle was slowly removed after an additional 5 min delay to prevent backflow and the wound was sutured. The rats recovered from surgery in a cage containing food and were heated by an incandescent light bulb.

***Collection and processing of umbilical cord blood***

Samples of UCB were collected from full-term placenta of healthy women, nonsmokers, nondrinkers, age ranging from 20 to 40 years, regardless of ethnic group at the Obstetric Service of Ghaem Hospital related to Mashhad University of Medical Sciences. Blood was collected in standard blood collection bags containing citrate phosphate dextrose adenine (CPDA) with 20-gauge syringe. UCB samples were diluted in a proportion of 1:1 in a phosphate-buffered saline solution (PBS) without Ca+^2^ and Mg+^2^.

***Isolation of*** ***umbilical cord blood mononuclear*** ***cells by the standard density gradient technique and BrdU labeling ***

UCB samples were transferred to centrifuge tubes (15 ml) containing Ficoll-Paque solution ( in a proportion of 8:3) and submitted to centrifugation at 800 g for 20 min in room temperature (RT) in order to isolate low-density umbilical cord blood mononuclear cells (UC-MCs). Mononuclear cells cloudy interface layer (buffy coat) was carefully removed by pipetting and transferred to a new tube and washed twice with PBS through centrifugation at 800 g for 10 -20 min (21), then the cells were resuspended in 1 ml of separated serum of UCB and were counted. Also their viability was enumerated by hemocytometer using trypan blue. The nucleated cells were, then, seeded into each tissue culture flask in RPMI medium supplemented with fetal bovine serum (10%) and 10 ml/l antibiotic. Then 3 µg, bromodeoxyuridine (BrdU), which labels DNA during the S phase, was added to every 1 ml of cell suspension and cells then were incubated at 37 ºC, humidified atmosphere containing 5% CO_2_ for 24 hr.

***Flow Cytometry***

UCB is a rich source of hematopoietic stem and progenitor cells (22, 23) which can express cell surface markers of CD_45_ and CD_34_ (24). So specific monoclonal antibodies anti-CD_45_–FITC, anti-CD_34–PE_ and the isotype control antibodies (immunoglobulin G1 [IgG 1]) were used for confirmation of existence of these cells. Thus 100 µl of the sample and 10 ml of each antibody were incubated for 20 min at RT in the dark. Erythrocyte lysis was performed by lysing solution of ammonium chloride.

Ten thousand cells were counted for each preparation on a FACS calibur fluorescence activated cell sorter (Beckton-Dickinson), and data analysis was performed with WinMDI 2.8 software. The flow cytometer was properly aligned, and fluorochrome compensation for fluorescein isothiocyanate (FITC) and phycoerythin (PE) was correctly tuned with respect to signal amplification. A gating strategy that uses light scattering parameters and CD_34_/CD_45_ fluorescence aided in accurate identification and enumeration of them.

***Administration of umbilical cord blood cells***

Twenty-four hr after surgery, behavioral tests were performed and animals were then randomized into four groups: Treated group 1 (n=10) received 1 ml suspension of UCBCs (4 ×10^6^) slowly for 5 min via the tail vein, treated group 2 (n=10) received 1 ml suspension of UCBCs (8 × 10^6^) intravenously (IV), lesion group (n=10) received intravenous 1 ml saline solution and control group (n=10) that did not receive any cells and solution.

***Functional Assessment***

Behavioral tests were performed for all 40 rats the day before ICH and also on days 1, 7 and 14 after ICH. These tests included limb-placement test (5, 25) and corner turn test (26, 27). 

The limb placing was performed on hand-held, immobile rats and involved separate tests for forward extension, lateral abduction, and adduction. For visual limb placing, a rat was slowly lowered toward a table top and held 10 cm above it with free hanging forelimbs. Normal rats reached, stretched, and placed both forepaws on the table top. By moving the rat laterally toward the table edge, lateral as well as forward visual limb placing could be assessed. Tactile forward and lateral limb placing were tested by lightly contacting the table edge with the dorsal or lateral aspect of a rat's paw, respectively. Proprioceptive limb placing involved pushing the rat's paw against the table edge to stimulate limb muscles and joints. Visual and tactile contact with the table was avoided by supporting the rat's chin and holding its head 45° upwards. Each rat was also put along the edge of an elevated platform in order to test proprioceptive adduction. A paw was gently pulled down and away from the platform edge and, upon sudden release, was checked for retrieval and placing. For each test, limb placing scores were 0, no placing; 1, incomplete and/or delayed (more than 2 seconds) placing; 2, immediate and complete placing. The highest score of 16 is typically given to normal rats.

In the corner turn test the rats were allowed to proceed into a corner, the angle of which was 30 degrees. To exit the corner, the rats could turn either to the left or the right randomly and this was recorded. This was repeated 10 times, with at least 30 sec between each trial, and the percentage of right turns was calculated. Turning movements that were not part of a rearing movement were not scored. 

***Histology and immunohistochemistry***

After performing behavioral test (14 days after surgery), rats were injected with an overdose of anesthesia (ketamine i.p.) and were then perfused through the ascending aorta (the cannula was inserted through the left ventricle) with the following solutions: 100 ml cold saline and then 100 ml of 4% paraformaldehyde in 0.1 mol/l phosphate-buffered saline. Then brains were removed and stored in 4% paraformaldehyde (pH 7.2) for at least 48 hr and were sectioned, after dehydration, into six equally spaced (2 mm) coronal blocks. The blocks were, then, embedded in paraffin. Every 25 µm coronal section was cut at a thickness of 6 μm between +0.1–0.86 mm of the bregma from each rat for a total of six blocks. These sections were used for H & E and immunochemical staining. The volume of lesion plus atrophy was calculated manually using an image analysis system [Image tool 3.0 (UTHSCSA)].

The volume of tissue lost= remaining volume of normal hemisphere-remaining volume of injured hemisphere

The volume of a hemisphere= average (area of the complete coronal section of the hemisphere - area of ventricle - area of damage) × interval between sections × number of sections.

The brain tissue residing between +0.1 and 0.86 mm of the bregma on the third block was the most severely injured and therefore the third block was specifically selected for immunostaining. After deparaffinization and rehydration of sections, endogenous peroxidase activity was blocked with 3% H_2_O_2_ for 10 min at 37 °C, then for DNA denaturation sections were placed in 2N HCl 30 min at 37 °C. Enzymatic retrieval was performed by trypsin solution then sections were blocked in a Tris-buffered saline containing 5% normal goat serum, 1% BSA and 0.05% Tween-20. Sections were then incubated with the monoclonal anti-BrdU as a primary (1:500) and anti-mouse IgG peroxidase conjugate as a secondary antibody (1:100) according to their datasheet. Subsequently for staining the cells diaminobenzidin (DAB) chromagen was applied. Negative control sections from each animal were prepared for immunohistochemical staining in an identical manner except for the primary antibodies which were omitted.

Counter were stains with Harris hematoxylin and cover slipped after imaging by BX51 microscope and 100X and 40X lense. To determine cellular co-localization of BrdU, an average of 10 histology slides of third block of brain per treated animals were evaluated. For computer-assisted counting, images were captured digitally to a monitor using the freely available software Image J (28). All BrdU-reactive cells containing a round or oval nucleus with intensely stained chromatin in the injured hemisphere were counted throughout all 10 coronal sections, whereas those with BrdU in the cytoplasm of macrophage-like cells were excluded. The purpose of the cell counts was not to estimate the total number of grafted cells but to obtain an accurate count within selected sections to compare among treated groups. 

***Statistical analysis***

Data were presented as mean±SEM. All behavioral and histological analyses were performed by researchers blind to group identity. Histological data and scores of functional tests were analyzed using analysis of variance. A *P*-value of < 0.05 was considered statistically significant.

## Results

***Flow cytometric analysis***

**Figure 1:**
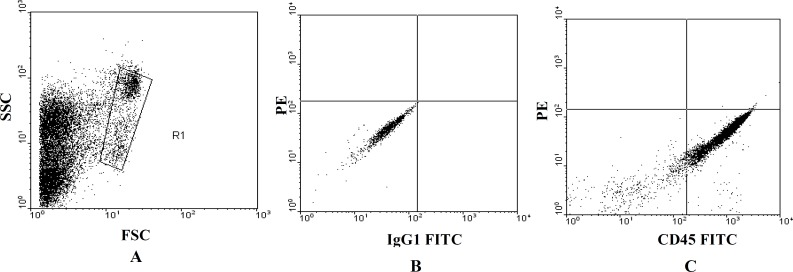
Flow cytometric analysis of umbilical cord blood-derived CD_45_^+ ^cells.

The analysis of the samples tested as shown in Figure 1 A, B, C indicate that the isolated mononuclear cells express cell surface markers of CD45 and the mean of percentage of gaited CD45+ cells is 89.43±0.43% (n=5) (Figure1 A, B, C).

(A) Gating of cells on a forward scatter (FSC)/side scatter (SSC) dot plot. (B) Evaluation of the frequency of non-specific antibody binding on a PE versus IgG1– FITC fluorescence dot plot of cells stained with control antibody. (C) Evaluation of the frequency of CD_45_^+^ cells on an PE versus CD_45_–FIITC dot plot of cells stained with anti- CD_45_ monoclonal antibody.

**Figure 2:**
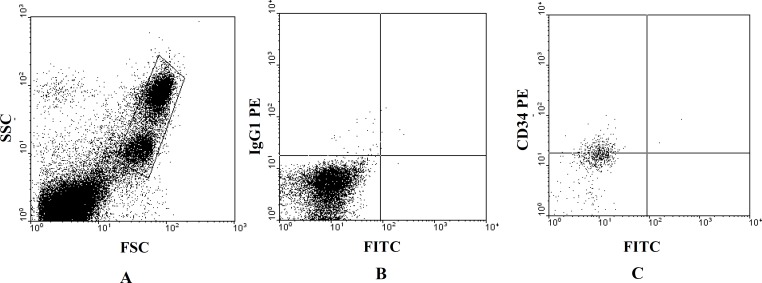
Flow cytometric analysis of umbilical cord blood-derived CD_34_^+^ cells.

(A) Gating of cells on a forward scatter (FSC)/side scatter (SSC) dot plot. (B) Evaluation of the frequency of non-specific antibody binding on a FITC versus IgG1– PE fluorescence dot plot of cells stained with control antibody. (C) Evaluation of the frequency of CD_34_^+^ cells on a FITC versus CD_34_–PE dot plot of cells stained with anti-CD_34_ monoclonal antibody.

Also analysis of the samples tested indicate that the isolated mononuclear cells express cell surface markers of CD34 and the mean of percentage of gaited CD34+ cells is 41.28% ± 0.13% (n=5) (Figure2 A, B, C).

***Functional Recovery***

In this study two neurological tests were performed for all groups at the day before ICH and on the days 1, 7 and 14 after surgery. The results of tests for three groups at the day before surgery were normal. But the tests outcomes after ICH showed that treated groups exhibit significant functional recovery compare with lesion group in 7 and 14 days after ICH. 

Limb placing test: In this test animals were examined for their ability to use the left and right forelimbs and hindlimbs. When rats were evaluated at day 1 after ICH, obtained scores in treated and lesion groups were not significantly different (8.2±0.2 and 8.2±0.13 vs 8.2±0.25, respectively). By day 7 after ICH, however, there was a significant improvement in treated rats in comparison with the lesion ones (12.7±0.26 and 13±0.3 vs 10±0.47, *P*< 0.05). This recovery was more prominent by day14 (14.9±0.31 and 15.2 ± 0.25 vs 11.9 ±0.53, *P* < 0.05). 

**Figure 3:**
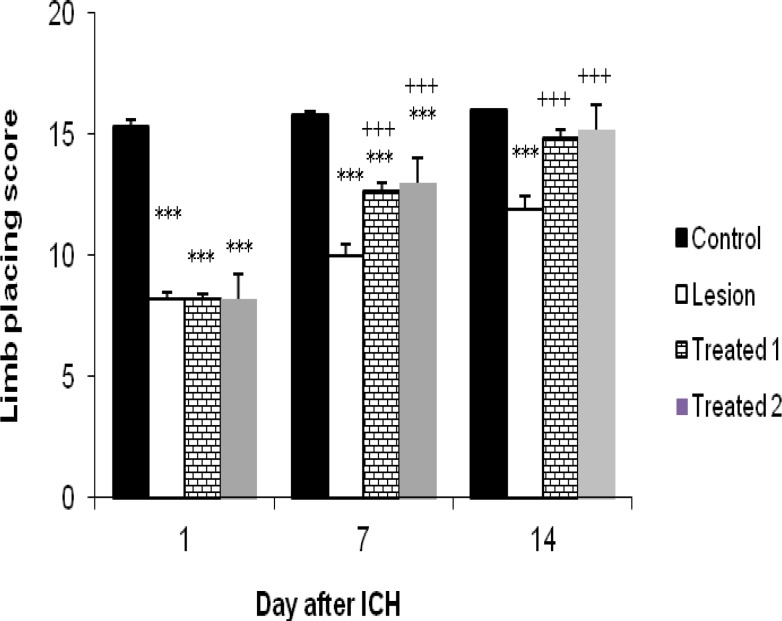
Effects of human umbilical cord blood infusion on limb-placing function in hemorrhagic rats.

Obtained scores in the control group receiving normal saline only instead of collagenase, at day 1 were significantly different (15.3±0.26, P< 0.05) from three others group. Although this significant difference continued by day 7 (15.8±0.13, P< 0.05), but at day 14, obtained results were different. In this manner, scores in this group were significantly different (16±0.0, P < 0.05) from lesion group (11.9±0.53) but there are no differences between this group and the treated groups (16±0.0 vs 14.9±0.31 and 15.2 ±0.25). So this time, the treated animals were near normal in their placement responses (Figure 3). Interestingly we didn’t observe any significant differences between obtained scores from two treated groups (*P*>0.05).

Animals treated with cord blood showed progressive improvement in limb-placing function over the 14 days of testing. Treated animals exhibited significantly better limb- placing performance (score±SEM) compared to lesion animals at 7 and 14 days after hemorrhagic brain injury (*P*< 0.05).  ***P< 0.001 vs control;* +++P< *0.001 vs lesion

**Figure 4:**
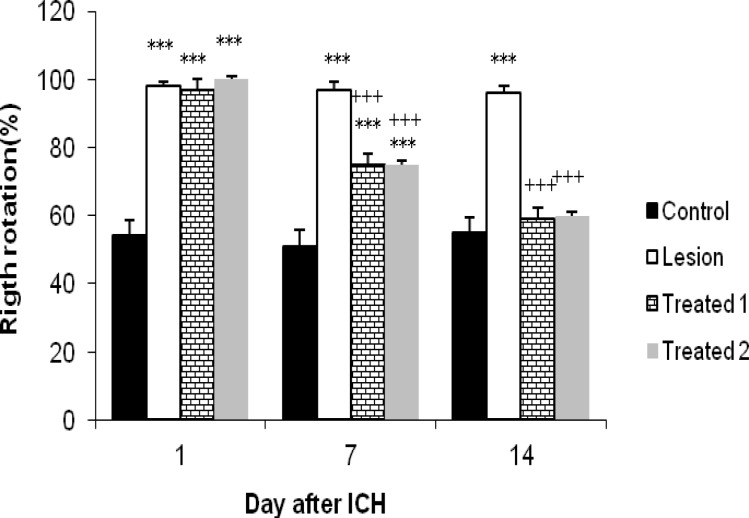
Effects of human umbilical cord blood infusion on corner-turn test in hemorrhagic rats.

Animals treated with cord blood exhibited a progressive decrease in ipsilateral (right) turns over the 14 days of testing and their results approached to control animals. By day 14 there was a significant decrease in turning right between treated groups and lesion group *(P*< 0.05*).* ***P<0.001 vs contro .*+++P<*0.001 vs lesion

Corner turn test: In this test, rats were tested for vibrissae sensory, postural and motor asymmetries and the number of ipsilateral (right) turns was recorded from 10 trials for each test on the days of 1, 7 and 14 after ICH. At day 1 after ICH the obtained results from treated and lesion groups were not significantly different (97% ±3 and 100% vs 98%±1.3, respectively). But at day 7 treated groups in comparison with lesion group (75%±3.07 and 75%±3.4 vs 97%±2.13, *P*< 0.05) showed a significant decrease in the percentage of right turns and this significant difference continued by day14 (59%±3.14 and 60%±3 vs 96%±2.21, *P<*0.05). 

On the other hand, animals in the treated and lesion groups at day1 and 7 after ICH, showed a significant (*P<*0.05) increase in the right turns percentage compared with control animals [ (97%±3 and 100% and 98%±1.3 vs 54%±4.52) and (75%±3.07 and 75%± 3.4 and 97%±2.13 vs 51%±4.82), respectively]. However, 14 days after ICH results for treated animals were changed and the percentage of turning right did not express any significant difference in comparison with control animals (59%±3.14 and 60%±3 vs 55%±4.53). But for lesion group the result in this day was similar to previous days and the percentage of right turns significantly differed from the results of control group (96%±2.21 vs 55%±4.53, *P< *0.05) (Figure 4). Similar to limb placing results did not cause any significant differences between obtained scores from two treated groups. (*P*> 0.05)

**Figure 5:**
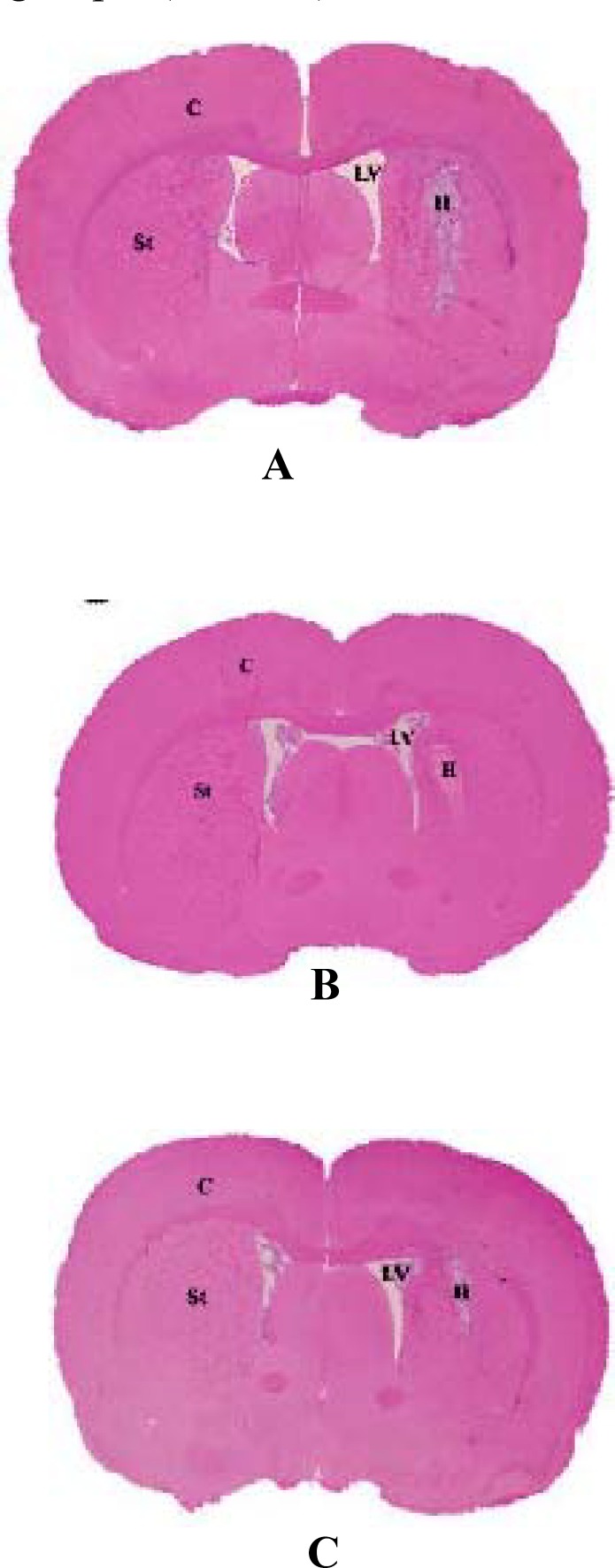
H&E staining showing sections of lesion group (A), treated 1 group (B) and treated 2 group (C).LV= Lateral ventricle. St= Striatum. H= Hematoma area. C= Cortex

***Volume of tissue loss***

**Figure 6:**
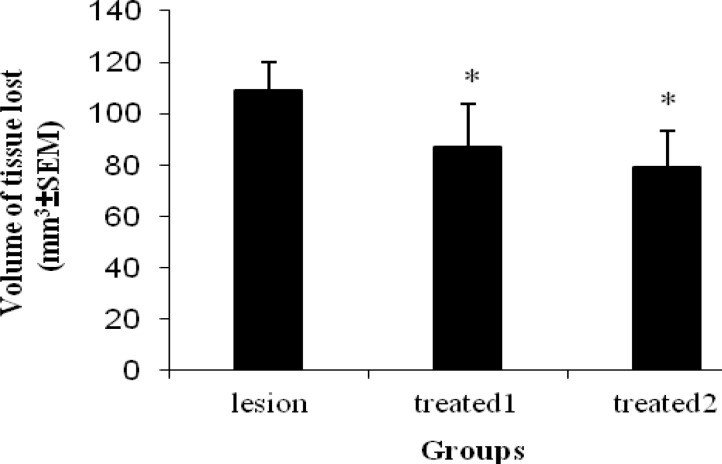
Quantitative striatal tissue loss volume, two weeks after ICH in treated and lesion groups. By day 14 there was a significant decrease in volume of tissue loss between treated groups and lesion group (*P< *0.05). *P< 0.001 vs lesion

The area of striatal tissue loss on the side of hemorrhage two weeks after ICH in treated and lesion groups is illustrated in Figure 5. The injured volume was considerably greater in the group of lesion (108.8±11.2 mm^3^) than the treated 1(96.7±17.2 mm^3^) and treated 2 (89.2±14.6 mm^3^). Therefore, the volume of striatal tissue loss was significantly (*P<*0.05) reduced in the treated groups when compared to the lesion group and this was illustrated in Figure 6. However, there was no statistically significant decrease of the lesion size between two treated groups. 

***Distribution and migration of HUCBCs in the injured brain***

**Figure 7:**
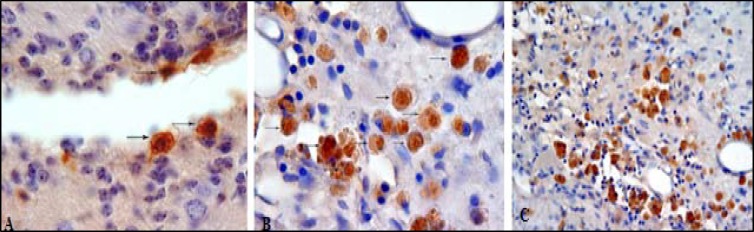
Histologic images of infused human umbilical cord blood in brain of treated groups (A to C).

In UCBCs treated animals, BrdU+ cells migrated selectively to the hematomal area in the right striatum and the light microscopic images on immunoperoxydase preparation were used to identify BrdU positive cells include small and round or oval nucleus with intensely stained brown chromatin (Figure 7). 

**Figure 8:**
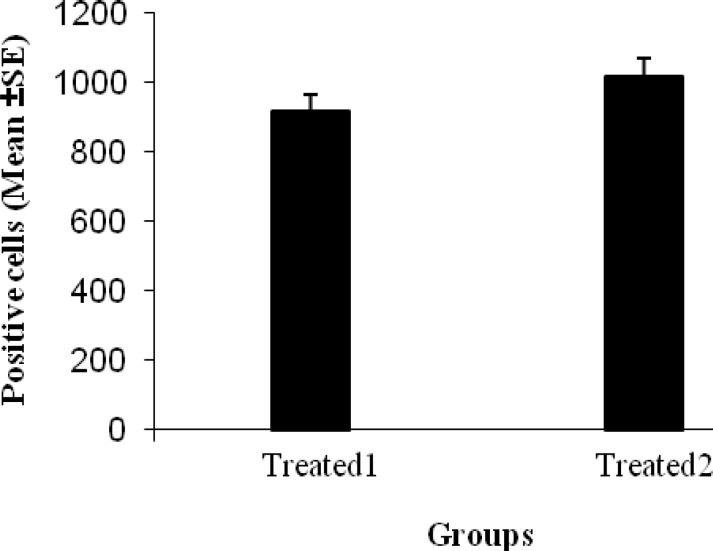
Mean of the number of BrdU+ UCBMCs (mean±SEM) in two treated groups that are not statically different from each other (*P*> 0.05).

The number of migrated BrdU positive cells at 14 days after ICH in first and second treated groups was 915.6±190 and 1017.6±228.1 cells/mm^2^, respectively (Figure 8) and there were not any significant differences between these two treated groups. 

Using immunoperoxidase with 3, 3-diaminobenzidine (DAB, brown) and counterstaining by hematoxylin for coronal brain sections. BrdU positive cells that are present in the residue of hemorrhagic area have round or oval shaped and brown nuclei and irregular cytoplasm. Arrows in A show the BrdU positive cells are migrating to the hemorrhagic area inside the parenchyma of the right striatum and arrows in B show the BrdU positive cells were settled inside the hemorrhagic area. Image of C, shows immigrants cells scatter inside the striatum. (Magnification is 100× for images A & B, and is 40X for C).

## Discussion

Knowledge about stem cells is rapidly growing and has recently opened new windows for brain repair strategies in acute and chronic neurodegenerative diseases. Progress in research area gives hope that new therapeutic options using stem cell transplantation could be used against brain tissue damage from ICH (29). A number of reports have been issued concerning stem cell transplantation in ICH. The categories of stem cells used for ICH treatment are neural stem cells (NSCs) (5, 30), embryonic stem cells (ESCs) (31), human mesenchymal stem cells (MSCs) (32), human bone marrow stromal cells (BMSCs) (33, 34), adipose-derived stem cells (ASCs) (35) and human umbilical cord blood cells (HUCBCs) (6, 36, 37).

The original clinically appealing feature of cord blood was the high concentration of hematopoietic stem cells, which is similar to that found in bone marrow. The potent hematopoietic activity of cord blood derived CD_34_^+^ cells may be attributed to the fact that cord blood is a much more developmentally immature source of stem cells in contrast with stem cells derived from adult sources (38). This promise is based not only on the evidence that HSC transplantation could be beneficial in the repair of tissue injury, but also on the fact that HSC are poorly recognized by HLA-incompatible hosts due to their limited immunogenicity (9), which allows them to be used in allogenous or xenogenous conditions. 

In the first report issued related to HUCBCs transplantation in ICH rat models, Nan *et al* found that intravenous infusions of umbilical cord blood can ameliorate the neurologic deficits associated with hemorrhagic brain injury (36), by using experimental pattern similar to ours, which involved the transplantation of HUCBCs intravenously at 24 hr after ICH, a collagenase model, and 2.4 to 3.2 million cells/rat. Particularly, it was found that animals receiving HUCBCs, recovered 6-13 days after ICH (stepping test, neurologic severity scale, and elevated body swing test). They also noted a scant number of human cord blood cells within the perilesional brain parenchyma despite the functional recovery observed. Finally they cocnluded that mechanisms other than cell replacement may underline the recovery effects of cord blood cells.

It is worthy to mention that in a similar study using cord blood in model of ischemic stroke Vendrame *et al* (13), at 4 weeks after infusion, observed a significant recovery in behavioral performance when 10^6^ or more HUCBC were delivered. Also infarct volume measurements revealed an inverse relationship between HUCBC dose and damage volume, which reached significance at the higher HUCBC doses. Moreover, HUCBC were localized by immunohistochemistry and PCR analysis only in the injured brain hemisphere and spleen. Their obtained results could complete the result of previous study by Nan *et al *(36).

After a while, Liao *et al* indicated that intracerebral administration of UC-MSC could accelerate neurological function recovery of ICH rat (6). In their study, an intracerebral hemorrhage (ICH) rat model was established by injection of bacterial collagenase VII and CM-DiI labeled human umbilical cord tissue derived mesenchymal stromal cells (UC-MSC) were intracerebrally transplanted into rat brain 24 hr after ICH and Morris water maze test was performed for neurological evaluation modified neurological severity score (mNSS). The results demonstrated that UC-MSC treatment significantly improved neurological function deficits and decreased injury volume of ICH rats.

In a similar study, researchers injected hUCMSC-Wharton's jelly stem cell to experimental model of traumatic brain injury (39) and they found statistically significant improvement in functional outcome in treatment groups compared with control group. Also, their histological finding exhibited significant increase in numbers of BrdU immunoreactive cells in traumatic core compared with other labeled group.

Since, recent studies focused on the whole HUCB and UC-MSC respectively, in the present study, mononuclear cells were isolated from HUCB and since, BrdU labeling technique is one of the most widely used technique for stem cell labeling in stem cell transplants fields (40), these cells were labeled with BrdU and then delivered to ICH rats intravenously with dose of 4 and 8 million cells/rats. The obtained results showed that firstly these HUCB-derived mononuclear cells can express both CD_34 _and CD_45_; a fact which is a strong evidence for being UC-HSC. Secondly, in this study we demonstrate that intravenous human umbilical cord mononuclear cells can improve neurologic deficits associated with intracerebral hemorrhage. Animals treated with cord blood in low and high dose exhibited improvements in limb placing test and corner turn test. At week two after HUCBC infusion, the majority of the behavioral measures demonstrated that when 4×10^6^ or more HUCBC were used after ICH there was significant recovery in behavioral performance. Interestingly, at the higher HUCBC doses used (8 ×10^6^), there was no further behavioral recovery. These results are consistent with similar studies that have utilized cord blood to treat hemorrhagic brain injury (6, 36, 37).

Thirdly, tissue loss was also significantly reduced in the treated groups compared with control group. But there is not any significant difference between two treated groups. Accordingly, immunostaining for BrdU marker illustrated significantly BrdU positive cells and enhanced stem cell migration in the perilesional region of the treated groups. So in spite of Nan’s results, mononuclear cord blood cells are shown to reduce the hematoma area and present study showed that, intravenously delivered HUCBC found in the brain 2 weeks after ICH were exclusively localized to the hemorrhagic hemisphere. The most convincing explanation is that the intravenously injected HUCBC may follow homing signals that attract them to the injured site. In fact, *in vitro* studies have shown that HUCBC can follow chemotactic signals from brain homogenates of stroke rats (16, 41) and a number of *in vivo* studies have described tendency properties by intravenously delivered cells (42, 43).

Formerly, cord blood mononuclear cells have been reported to express angio-genic factors such as vascular endothelial growth factor and angiopoietin-1 and 2 (44) so obtained results from decrease in lesion size in our study suggest that HUCBC infusion could be mediating functional recovery from ICH at the level of the cerebrovasculature. Moreover, after a hemorrhagic insult, a cascade of inflammatory molecular and cellular events takes place and clinical studies have suggested that this acute response affects not only clinical outcomes but also the extent of brain injury (45). Therefore, HUCBC treatment may influence the cascade of inflammatory/immune events and thereby explain the neurobehavioral and histological benefits observed in this study. The mononuclear fraction of cord blood cells produces large amounts of IL-10 (46), a potent anti-inflammatory cytokine, which may be involved in reducing the post hemorrhagic inflammatory response. Molecular-based mechanisms may include not only immune processes mediated by interleukins but also the involvement of growth/trophic factors. The mononuclear cord blood cells have been shown to express growth factors, such as nerve growth factor (47) and some investigators have hypothesized the major role of endogenous neuroprotective factors in the HUCB-induced ischemic brain recovery (48).

## Conclusion

In summary, we demonstrated that intravenous administration of mononuclear cells of human umbilical cord blood was functionally and histologically beneficial to rats after ICH and transplanted UC-MCs can improve neurological function recovery of ICH in rat and decline the striatum lesion size. Regardless of the mechanism of action, the clinical relevance of this HUCBC-based therapeutic option for hemorrhagic stroke is obvious, although additional work is needed before use of HUCBC in the clinical trial.
